# Factors affecting motivation of close-to-community sexual and reproductive health workers in low-income urban settlements in Bangladesh: A qualitative study

**DOI:** 10.1371/journal.pone.0279110

**Published:** 2023-01-13

**Authors:** Ilias Mahmud, Sumona Siddiqua, Irin Akhter, Malabika Sarker, Sally Theobald, Sabina Faiz Rashid

**Affiliations:** 1 Department of Public Health, College of Public Health and Health Informatics, Qassim University, Al Bukairiyah, Saudi Arabia; 2 BRAC James P Grant School of Public Health, BRAC University, Mohakhali, Dhaka, Bangladesh; 3 Heidleberg Institute of Global Health, Heidelberg University, Heidelberg, Germany; 4 Department of International Public Health, Liverpool School of Tropical Medicine, Pembroke Place, Liverpool, United Kingdom; Indian Institute of Technology Jodhpur, INDIA

## Abstract

Close-to-community (CTC) health workers play a vital role in providing sexual and reproductive health services in low-income urban settlements in Bangladesh. Retention of CTC health workers is a challenge, and work motivation plays a vital role in this regard. Here, we explored the factors which affect their work motivation. We conducted 22 in-depth interviews in two phases with purposively selected CTC health workers operating in low-income urban settlements in Dhaka, Bangladesh. We analyzed our data using the framework technique which involved identifying, abstracting, charting, and matching themes across the interviews following the two-factor theory on work motivation suggested by Herzberg and colleagues. Our results suggest that factors affecting CTC sexual and reproductive health workers’ work motivation include both extrinsic and intrinsic factors. Extrinsic or hygiene factors include financial incentives, job security, community attitude, relationship with the stakeholders, supportive and regular supervision, monitoring, and physical safety and security. While, the intrinsic factors or motivators are the perceived quality of the services provided, witnessing the positive impact of the work in the community, the opportunity to serve vulnerable clients, professional development opportunities, recognition, and clients’ compliance. In the context of a high unemployment rate, people might take a CTC health worker’s job temporarily to earn a living or to use it as a pathway move to more secure employment. To maintain and improve the work motivation of the CTC sexual and reproductive health workers serving in low-income urban settlements, organizations should provide adequate financial incentives, job security, and professional development opportunities in addition to supportive and regular supervision.

## Background

More than 35% of the urban population of Bangladesh lives in low-income, and often informal, settlements [1]. The population in these settlements is growing by 7% annually, mainly because of an influx of rural migrants [1]. Current public health services are inadequate to satisfy the healthcare needs of this growing population. This vacuum in health services is mainly filled by informal and poorly regulated private health service providers with dubious quality [2]. However, a few non-government organizations (NGOs) also operate in these settlements through Close-to-Community (CTC) health workers [2]. CTC health workers are a group of heterogeneous providers of health services at the community level and known by different titles in different country contexts, such as community health workers, community health volunteers, community distributors, community health nurses, community-directed health workers, health auxiliaries, health promoters, family welfare educators, village health workers/volunteers/teams, community health aides, barefoot doctors, traditional healers, practitioners who combine traditional and modern medicine, allopathic practitioners, drug sellers and faith healers [3].

CTC health workers play a vital role in achieving universal health coverage. They are instrumental in providing cost-effective and efficient delivery of primary healthcare in low- and middle-income countries (LMICs) [4–6]. They link the formal health systems to the community that otherwise would remain beyond the reach of formal health facilities [7]. CTC health workers, both formal and informal, are an important part of our health system in mitigating the challenges of the shortage of health professionals [8]. Yet, they remain particularly vulnerable to de-motivation and, consequently, high rates of attrition [9,10]. This puts a considerable strain on our health systems, due to the cost and time necessary to recruit and train new health workers [11].

Motivation is an important factor in preventing health worker attrition [12,13], burnout [14], turnover [15], and promoting better quality services [16]. The motivation of the CTC health workers is associated with both individual and programme performance [17,18]. Understanding the factors affecting the motivation of CTC health workers is important because community health programmes’ success and failure depend on their performance [19]. Effective management, training opportunities, and supportive supervision along with both financial and non-financial incentives are needed to keep them motivated, performing, and retained for a longer term [20,21].

CTC Sexual and Reproductive Health (SRH) workers provide promotional, preventive, and curative SRH services, including menstrual regulation, in low-income urban settlements in Bangladesh and experience even greater challenges because of the sensitivity of SRH issues [22]. Hence, it is particularly important to understand the factors which motivate or demotivate them to continue their job for the improvement of community SRH services in low-income urban settlements. This paper reports the factors affecting the motivation of CTC SRH workers operating in low-income urban settlements in Bangladesh.

## Methods

This study is a part of a larger project on improving CTC SRH services in low-income urban settlements in Bangladesh [22] and relied on a qualitative approach. We conducted 22 in-depth interviews with purposively selected CTC SRH workers (Table 1) using semi-structured interview guidelines. CTC SRH workers we interviewed included 14 salaried workers and eight volunteers who receive a little monetary incentive based on their performances. We recruited CTC SRH workers from both the NGO and Government sectors. We conducted our in-depth interviews in two phases. In the first phase, we interviewed 10 CTC SRH workers and in the second phase, we did another 12 interviews. First-phase interviews were intended to understand the overall challenges of the CTC SRH workers including their motivation. While the second phase of interviews exclusively explored the factors affecting their motivation. We have conducted interviews at the participants’ residences or workplaces based on their convenience.

**Table 1:** Characteristics of the participants.

No	Age	Gender	Designation	Job-status	Job description	Experience	Educational attainment
1.	27	Female	Health Educator	Salaried	Door-to-door visits, pharmacy, diagnostic center, and other clinic visits, ANC, PNC, and MR counseling and referral, inform community people about satellite clinics and note their regular work in the official register. Supervised by the Clinic Manager	4 months	Masters
2.	45	Male	Field Coordinator	Salaried	Pharmacy, diagnostic center, and other clinic visits, ANC, PNC, and MR counseling and referral, maintain their regular work in the official register. Supervised by the Clinic Manager.	3 years	Masters
3.	32	Male	Health Educator	Salaried	Previously mentioned	1 year 6 months	Masters
4.	28	Male	Outreach worker	Salaried	Door-to-door visits, pharmacy, diagnostic center, and other clinic visits, ANC, PNC, and MR counseling and referral, inform community people about satellite clinics, note their regular work in the official register, and supervise volunteers. Supervised by a service promoter.	6 months	Masters
5.	32	Female	Marie Stopes Volunteer	Volunteer	Attending satellite clinics and counseling community people for taking services from satellite clinics. Refer people to satellite clinics and static clinics. Supervised by an Outreach Worker.	4 months	Primary
6.	32	Male	Field Coordinator	Salaried	Previously mentioned	8 years	Bachelor
7.	50	Female	Marie Stopes Volunteer	Volunteer	Previously mentioned	6 months	No formal education
8.	33	Female	Service Promoter	Salaried	Door-to-door visits, pharmacy, diagnostic center and other clinic visits, ANC, PNC, and MR counseling and referral, inform community people about satellite, note their regular work in the official register, supervise Outreach Worker and MSV and supervised by a Clinic Manager.	5 years	Masters
9.	22	Female	Service Promoter	Salaried	Previously mentioned	2 years 6 months	Higher Secondary
10.	44	Female	Service Promoter	Salaried	Previously mentioned	19 years	Bachelor
11.	22	Female	Peer Educator	Volunteer	Attend community awareness-raising programmes, peer education, and referral.	4 months	Primary
12.	35	Female	Peer Educator	Volunteer	Mentioned previously	1 year	No formal education
13.	31	Female	Paramedic	Salaried	Give services and counseling for general health problems, ANC, PNC, MR, PAC & normal delivery at static clinic and satellite clinic. Supervised by a Clinic Manager	-	Secondary
14.	30	Female	Community Health volunteer	Volunteer	During monthly household visits, they provide health promotion sessions and educate families on nutrition, safe delivery, family planning, immunizations, hygiene, and water and sanitation. They also use this time to sell health products, such as basic medicine, sanitary napkins, and soap.	-	Primary Incomplete
15.	43	Female	Family welfare visitor	Salaried	Give general health and family planning services at union health and family welfare centers throughout Bangladesh	2 years	Higher Secondary
16.	40	Female	Family welfare visitor	Salaried	Mentioned previously	-	Masters
17.	42	Female	Family Welfare Assistant	Salaried	Trained to screen and offer a variety of services including family planning, support for pregnant women, TB case detection and treatment support, and immunizations among others.	-	Secondary
18.	45	Female	Community Health volunteer	Volunteer	Previously mentioned	2 years	Primary
19.	38	Female	Community Health volunteer	Volunteer	Previously mentioned	-	No formal education
20	25	Female	Community Health volunteer	Volunteer	Previously mentioned	-	Higher Secondary
21.	25	Female	Family welfare visitor	Salaried	Mentioned previously	-	Secondary
22.	41	Female	Family welfare assistant	Salaried	Mentioned previously	-	Secondary

All interviewers had a university degree in a social science discipline with prior experience in qualitative research. In addition, they received training on qualitative interviews and ethical issues specific to this research. Interviews were conducted in the local language (Bangla) and were recorded with an audio recorder. All the participants gave their written informed consent for the interviews.

Interviews were transcribed verbatim in Bangla by the respective interviewers. All transcripts were then translated into English by a bilingual professional translator with prior experience in translating qualitative interviews. The authors read each interview transcript several times to identify the factors positively or negatively affecting the motivation of CTC SRH workers. We used the framework technique to analyze our data [23]. This technique involved identifying, abstracting, charting, and matching themes (in our case, factors affecting the motivation of CTC SRH workers) across the interviews following the two-factor theory on work motivation suggested by Herzberg and colleagues [24]. All identified statements were considered, but statements were consolidated either into intrinsic factors, also known as motivators, or extrinsic factors, also known as hygiene factors, as Herzberg and colleagues suggested in their two-factor theory on work motivation [24] where it was perceived that the underlying factors were the same. The intrinsic factors or motivators are associated with the need for growth or self-actualization. These factors included achievement, recognition, the work itself, responsibility, advancement, and the possibility for growth. Whereas the extrinsic factors or hygiene factors are related to the need to avoid unpleasantness and included company policies and administration, relationship with supervisors, interpersonal relations, working conditions, and salary [24].

## Results

To explore the factors which affect the motivation of CTC SRH workers in low-income urban settlements in Bangladesh, we employed a qualitative approach to data collection and analysis. Our findings suggest that the motivation of CTC SRH workers is influenced by both extrinsic and intrinsic factors (Table 2).

**Table 2:** Empirical constructs of motivation in CTC SRH workers in low-income urban settlements in Bangladesh.

Extrinsic/hygiene factors	Intrinsic factors/motivators
**Community factors**	**Perceived value in the work**
Community attitudes toward CTC SRH workers	Perceived quality of the SRH services provided
Relationship between CTC SRH workers and the community	Opportunity to serve vulnerable women
Perceived safety and security in the community	Perceived impact of the work on clients’ lives
**Organizational factors**	**Professional growth**
Relationship with co-workers and supervisors	Opportunity to improve knowledge and skills
Supervision	Promotion opportunities
Workload/target	
**Financial stability factors**	**Recognition**
Remuneration	Recognition from the community, clients
Job security	Recognition from supervisors
Retirement benefits	Clients’ compliance

## Extrinsic factors

Extrinsic or hygiene factors, we identified, are grouped under community, organizational and financial stability factors.

### Community factors

Community factors included community attitudes towards CTC SRH workers, the relationship between CTC SRH workers and the community, and perceived safety and security in the community.

#### Community attitudes and relationship with the community

CTC SRH workers mostly reported that the community has positive attitudes towards them and their services. They appreciate the need of having positive community attitudes in carrying out their job duties. Hence, they put a conscious effort into developing and maintaining positive community attitudes toward them and their activities. In this regard, a CTC SRH worker told, *“most of the people*, *almost 90% of them*, *behave well*.*”* (Field coordinator, male, 35-year-old). While another further elaborated and said, *“Local people have positive attitudes toward me*. *For example*, *when I walk through the road*, *they catch me and start to talk about their problems*, *or just start social chatting*. *Sometimes*, *I am time pressured*, *but I must talk to them*. *I feel*, *if I do not hear them today*, *they will not come to me tomorrow for my services; they will go elsewhere*. *That’s why I pay attention to them [even when I am busy]*.*”* (Family welfare visitor, female, 43-year-old).

The job of CTC SRH workers requires them to meet and interact with people in the community. Some workers find it enjoyable. For example, a health educator said, *“I am satisfied [with my job]*. *I like talking to people… [Because of my job] I can meet new people and get acquainted with them*. *Sometimes*, *I find people who have migrated from my village”*. (Female, 27-year-old).

On contrary, CTC SRH workers also experience negative behaviours from the community people which demotivates them. Often this is because of the stigma associated with SRH issues. For example, a female volunteer said *“Many do not like us discussing SRH issues with their adolescent children*. *Sometimes some people misbehave with me when I visit their house*. *I feel sad at that time*.*’* (Community health volunteer, 32-year-old).

#### Perceived safety and security in the community

Sometimes, CTC SRH workers are concerned with their safety in the field. They feel the need for a partner while working in the community. Concern with own safety works as a demotivating factor. In this regard, a female service promoter said, *‘working alone is a boring thing*. *If I get a man or woman work partner*, *it will help me to do my work better and increase my client number*. *Moreover*, *working alone is risky for women*. *In some areas*, *people are not good*. *Sometimes/some places*, *where a man can enter*, *a woman cannot*. *In this case*, *if someone accompanies me*, *I feel secure*, *and it also helps to do my work better”*. (Service Promoter, 22-year-old). Safety is not a concern only for female workers. A male health educator said, *“The most challenging is slum [informal settlements] visits*. *I can enter a pharmacy at any time*, *but I cannot enter a slum at any time…I am a man; it is risky for me to enter a slum at any time*. *It is risky for women as well*.*’* (Health educator, 32-year-old).

### Organizational factors

#### Relationship with co-workers and supervisors

Most of the CTC SRH workers, we interviewed, mentioned the working environment as good and motivating. They were coming timely to the office and going to the field regularly. They like to have a good bonding with co-workers, and openly discuss their field experiences, problems, comforts, and discomforts with them. One participant stated, *“There are seniors and juniors*, *but we all are like family here*. *I can talk to everyone openly*, *and pleasantly*. *This is a good environment*. *Our supervisors behave well with us and honour us*. *We also respect them*. *We have noticed that in some organizations it is necessary to address your supervisor as boss or sir*. *Here*, *we address them as brothers*, *and they also address us as brothers*. *So*, *a cordial work environment is developed”*. (Field coordinator, male, 35-year-old).

Another CTC SRH worker said, *“All of my colleagues are good*. *We help each other in work*. *We can discuss with each other when there are issues [challenges]*, *even personal family issues*. *Therefore*, *we get motivation in our work*.*”* (Health educator, female, 27-year-old).

We found that CTC SRH workers help each other when they experience challenges. They help each other to achieve their monthly job target. This support and cooperation between them work as a motivating factor. A CTC SRH worker mentioned, *“I like the environment*. *I like the cooperation I receive from my colleagues*. *I like their behaviour*. *That’s why I like to work here*.*”* (Health educator, male, 32-year-old). In this regard, another told that “*Reza [pseudonym] is a very good colleague*. *When we are short of the daily target [number of clients]*, *he tries to work harder and say*, *“Let’s go sister; let’s visit more*. *Today the number of clients is less*.*”* (Volunteer CTC SRH worker, female, 50-year-old). Therefore, CTC workers work better due to the friendly and supportive working environment. Appreciation is one of the key factors to enthuse to work better.

#### Supervision

We found that supportive supervision and regular supervision motivate CTC SRH workers. They reported that their supervisors always help them when they face difficulties in the field. They immediately contact their supervisors when they need their suggestions and decisions. The data suggest that supportive and regular supervision helps in three ways- first, by improving CTC SRH workers’ skills, second, through supportive supervision supervisors can be positive role models and third, as a tracking mechanism of the performance of the CTC SRH workers.

CTC SRH workers reported that their skills have improved following supportive and regular supervision. Therefore, they feel good and motivated when they receive supportive and regular supervision. A CTC SRH worker mentioned, *“I did not understand my work well before*. *Because of her supervision*, *it became possible for me to take my work easily”*. *(Service promoter*, *female*, *22-year-old)*. Another CTC SRH worker, who joined her job 7 months ago, mentioned, *“She [my supervisor] is teaching me how to do my work better as I’m new*. *She is in the work for a long*. *She teaches me well and tells me ways of getting success in my job*. *This is how I get motivation from her*. *My previous supervisor was also very good*. *She used to visit the field with me and used to correct me when I did any mistake while talking to people*. *She used to guide us to better attract the attention of people through our talk”*. (Health educator, female, 27-year-old).

We also found that CTC SRH workers get inspired and motivated when their supervisor accompanies them in the field. It helps them to take instant decisions like providing subsidies to a patient in an emergency. Sometimes, local people create pressure on the CTC SRH workers to stop providing sensitive SRH services, such as menstrual regulation. In these cases, their supervisors can intervene during field visits- they talk to the local people including gatekeepers and politicians to solve the problems. Working in the field for a stigmatized issue like menstrual regulation is challenging. Hence, CTC SRH workers sometimes feel neglected when their supervisors do not visit the field because of their other workload.

We further found that regular supervision keeps the CTC SRH workers on track and motivates them to perform well, hence is appreciated. In this regard, a CTC SRH worker said, “…*if they leave us un-monitored and don’t keep any track*, *there will be a tendency of lagging behind*. *But*, *if the work is monitored regularly*, *for example*, *if my work is supervised everyday then a sense of accountability will work in me and this sense of accountability will motivate me in my work*. (Health educator, female, 27-year-old).

On the contrary, we found that unsupportive supervision demotivates CTC SRH workers. For example, a health promoter mentioned, *“I feel constant pressure from all around me*, *but still*, *I work very hard with a smile on my face…a few days ago our line manager insulted me in front of others*. *I was telling [people from other organizations] that I got this big opportunity as a women leader*. *He [my line manager] stopped me and said*, *‘what women leadership are you talking about; you have done this and that wrong’*. *He could have spared me from this insult*. *He could have provided me feedback in private as he is my supervisor*.*”*

In another instance, the same worker said: *“Well*, *friendliness is the main thing*, *not reproaching*. *My supervisors do not think of it like that…I have seen many times that many managers severely reproach supervisees even if they have done good work*. *They constantly criticize them*. *Criticism cannot make one skilled or stronger*, *but motivation can… I think the supervision process can improve skills… Does supervision mean controlling*, *no*!*”*

CTC workers prefer to be monitored based on meeting the targets. However, often they are monitored on maintaining office hours which works as a demotivating factor for some. For example, a community SRH worker mentioned, *“Well when they scold me*, *I feel demotivated*. *When they tell me ‘You are avoiding work too much; stop doing it*.*’ I become extremely careless sometimes (laughs)*. *Sometimes*, *I avoid the afternoon session because of my other involvements*. *They criticize me a lot for this*. *I wish I could tell them that it does not matter how long I have been in the field; check if I have done the work*, *check if there is any achievement or not*.*”*

#### Workload/Target

There are many drug stores and private clinics, which are often illegal, poorly regulated, and provide unsafe services, available in and around the community. CTC SRH workers face fierce competition from informal providers, such as drug store salespeople and unregulated or poorly regulated private clinics. In this regard, a worker said, *“Motivation increases when we get clients*. *It is normal to feel bad if we roam around in the community the whole day but can’t find a single client*. *If the target of ligation and other contraceptive methods can be met*, *we feel good*. *We feel bad if we can’t meet the target [set by the management]*.*”* (Family welfare assistant, female, 42-year-old). Even after putting in their best effort, when they receive negative feedback from their superiors for not meeting the target, they feel frustrated and demotivated. For example, a worker mentioned, *“I feel tired*, *demotivated*, *and depressed*. *I feel bad*. *It is felt worse if the target is not achieved even after doing so much hard work*. *On the other hand*, *the office also asks*, *what are you doing*?*”* (Outreach worker, male, 28-year-old).

Unrealistic targets, pressure to achieve the target and lack of appreciation from the superior for the effort community workers are putting in may result in poor job satisfaction and demotivation. A service promoter mentioned, *“I work hard*. *I must feel bad when you do not appreciate my effort*. *I feel constant pressure from all around me*.*”* (Service promoter, female, 33-year-old).

### Financial stability factors

#### Remuneration

Lack of perceived fair remuneration is one of the major factors of demotivation among the CTC SRH workers in informal urban settlements in Bangladesh. This is illustrated by the following quote: *“I am not satisfied with my salary*. *Besides*, *annual increment also depends on salary*. *Annual increment would be less if the salary is less*.*” (Field coordinator*, *male*, *35-year-old)*. Another outreach worker said: *“Encouragement is necessary for doing work*. *The amount they give us as salary is making it next to impossible to live in Dhaka*.*”* (Outreach worker, male, 28-year-old). Echoing the same a female volunteer said: *“I like this work; hence I am doing this*… *But our financial incentives are not justifiable considering our workload…If someone asks us about our financial benefits*, *we feel ashamed*. *They ask us “why you are doing this work with such a low salary”*.

In this regard, a field coordinator said: *“No benefits [increment or bonus] …it does not matter how well I work; how much I achieve*. *Even when I achieve my targets–there are no extra benefits [financial]*.*”* (Field coordinator, male, 32-year-old).

#### Job security and retirement benefits

Lack of job security and retirement benefits may demotivate CTC SRH workers in the low-income urban settlements in Bangladesh. This is evident in the following quotation: *“Many [office-based workers] are appointed as a permanent employee after two*, *three or four years of service*. *I am working here for eight long years*! *They are not appointing us as permanent staff*. *If I leave my job now*, *I will have to go with an empty hand*, *life is zero*.*”* (Field Coordinator, Male, 32-year-old).

### Intrinsic factors or motivators

#### Perceived value in the work

CTC SRH workers work in a pluralistic environment where there is fierce competition between different formal, including NGO and private providers, often with dubious quality, and informal providers of SRH services. This context makes the women in need of any sensitive SRH services vulnerable to unsafe practices and exploitation. Our data suggest that knowing that they are providing quality services to vulnerable women and observing the positive impact of their work on vulnerable clients’ lives inspire motivation among the CTC SRH workers.

#### Perceived quality of the SRH services provided

Our data suggest that the CTC SRH workers feel motivated when they perceive that the services their organization is providing are of good quality, hence helping people. For example, a health educator mentioned, “*I like helping people*. *The SRH services we provide here are of good quality*. *I like this very much*. *Therefore*, *I like working here*”. (Female, 27-year-old).

We also found that often CTC SRH workers themselves do not endorse certain contraceptive methods considering the perceived negative effect of such methods. Hence, they find that task demotivating. For example, the same worker mentioned, *“Sometimes there are IUD camps*, *and we must bring clients for IUD camps*. *But some of them [women in the community] don’t want IUD*. *They don’t like it*. *I too don’t feel good doing that*. *I feel that it would be better if we don’t have to do this…It makes problems*, *and many of them faced problems*. *It makes some of them bleed very much*. *As it remains for 10 years*, *so it can create problems… It has even caused the death of some people*. *Many people fear this contraceptive method as it can bring either a positive or a negative result*. *People are worried about the negative aspect*. *So*, *they are not going for it*.*”*

#### Opportunity to serve vulnerable women

We found that a feeling of satisfaction in serving vulnerable women keeps CTC SRH workers motivated to perform their job responsibilities. This is illustrated by the following quote: *“Everyone doesn’t get the opportunity to help people*. *I am lucky that God has given me this opportunity [to provide SRH services to vulnerable women]*”. (Service promoter, female, 22-year-old).

Another worker told, *“Well*, *a helpless girl*, *who cannot tell anyone that she has got pregnant*, *feels happy when she sees us*. *I feel good doing these types of work*. *I mean she cannot tell anyone but feels good to see us*. *These are the secrets [of my motivation]*.*” (Service promoter*, *female*, *44-year-old)*. Another said, “*If we can stand by a patient of our locality*, *isn’t it a matter of joy for us; our encouragement*?*”* (Peer educator, female).

#### Perceived impact of the work on clients

Our data also suggest that perceiving a positive impact of their work on the community and themselves keeps the CTC SRH workers motivated to carry out everyday job responsibilities. They like to work in low-income urban settlements to promote safe SRH services. Their main job responsibilities included identifying women in need of SRH services including menstrual regulation, providing SRH information related to prevention and treatment, linking them to a formal clinic, and following up and encouraging the women to receive care in a formal health facility. CTC SRH workers move around the community and spend a great deal of time in the community. Hence, they understand the vulnerability of women in need of menstrual regulation or other SRH services. Therefore, they feel good knowing that they are doing something good for these helpless women. One CTC SRH worker told, *“I am satisfied with my job*. *I never feel bad to work*. *I like to serve people*. *This is a valuable service- extending help to others in great need*. *People pray for me*. *I can approach them easily*. *Many people love me*, *like me*, *trust me*. *When they share their heart feelings with me considering me a near one of their lives*, *I feel honoured*. (Family welfare visitor, female, 43-year-old).

Helping women for receiving safe and affordable SRH services, particularly menstrual regulation, give these CTC health workers a sense of satisfaction. A female CTC SRH worker said, ***“****I try my best to help them [women in need of menstrual regulation]*. *I wish they come to me first instead of wasting thousands of taka [Bangladesh currency; in informal or poor-quality private clinics] by the influence of the middlemen*.*” (Service promoter*, *22-year-old)*.

### Professional growth

#### Opportunity to improve knowledge and skills

CTC SRH workers value the opportunity to learn new things after coming into this profession. They learn from the doctors, other health workers of the NGOs, or any partner organizations they are working with. In this regard, a field coordinator said *“I have learned everything after coming here; that’s why I am satisfied*. *I am learning many new things while serving clients [about their SRH issues and available treatment options]*”. (Male, 45-year-old).

Another worker said, *“I don’t become upset when they [supervisors] tell me to do things differently because they are teaching me…*. *They are instructing me because I don’t know enough*. *I have the desire to learn*. *I must learn this*. *I perform my duties at my best*. *I would be grateful [to my supervisors] if they could teach me completely and if I could learn from them completely*.*”* (Volunteer, female, 50-year-old).

#### Promotion opportunities

Promotion opportunities motivate CTC SRH workers. For example, a newly appointed, working only for four months, CTC SRH worker was expecting a promotion following a show of good work: *“It is great if I can do my work well*. *I would get a promotion if I can fulfill my job responsibilities”*. However, few workers reported that they don’t get any promotions and are tired of working in the same position. For example, a health educator mentioned, *“The major point is that no one wants to work at a lower position for a long time*… *For this [lack of promotion opportunity] it may not be possible for me to continue*. *If I get a better chance and get a good job*, *I will leave*. *Everyone wants quality*, *good position*, *and respect*.*”* (Health Educator, Female, 27-year-old).

#### Professional networking

Many community SRH workers have little formal education. However, their job enables them to make connections with doctors working in different hospitals and clinics in and around their neighbourhood. They often use this connection to take their clients to clinics or hospitals and negotiate a reduced service charge for them. In return, they earn respect from the community they are working for. This is hugely motivating for the community SRH workers. For example, a peer educator mentioned, *“I have taken quite a few patients there [a government hospital] … There was a woman nearby whose parents have died*. *She was very poor*. *Her delivery was due*. *She had two children*, *but both died*. *I knew Mitford [a government hospital]*, *so I took her there*. *I told the doctors there that she is very poor; her two children have died before; please take less money from her if you can*. *They gave her all the medicines she needed*, *and her baby was delivered there*. *It did not cost her much”*. (Peer educator, female, 35-year-old).

### Recognition

#### Recognition from supervisors

When supervisors praise their work, CTC SRH workers feel very inspired. For example, a CTC worker told, *“Supervisor compliments us if he likes our work*. *Then*, *he advises us on how to work better by improving our skills*.*”* (Paramedic, female, 25-year-old). In this regard, another motivated CTC worker said, *“We have the freedom*. *We work the way we want to*. *I do every work in my responsibility”*. (Family welfare assistant, female, 42-year-old).

#### Recognition from the community

CTC SRH workers maintain a good relationship with the community people including the gatekeepers and political leaders. Community people often praise them for the services they provide. They feel very proud when people praise their services. This keeps them going and inspires them to work harder for providing better SRH services. In this issue, one CTC SRH worker told that *“Their [community people’s] affection and respect for me encourage me to provide better service…We must hear everything [they tell] with attention*. *If I pay attention to them*, *they will say that I am good and generous to them*.*”* (Family welfare visitor, female, 43-year-old).

People living in these low-income and informal urban settlements mostly rely on the CTC SRH workers for their SRH needs, particularly for menstrual regulation. Hence, when any worker faces any problem during their field visit community people help them. It would not be possible for the providers to work in the field without the acceptance of the community people. In this regard, a CTC SRH worker mentioned, *“People in this area give me tremendous support*. *However*, *few people say that I refer people for my own sake*. *However*, *most people appreciate my services*. *I have good relationships with people*. *Good relations are helpful for my work too*. *Sometimes I must visit the community even at 3*:*00 a*.*m*.*…Serving people is my main concern*.*”* (Community health volunteer, female, 30-year-old).

#### Recognition from the clients

We found that a sense of being respected and valued by the clients motivates the CTC SRH workers. Many clients in the informal urban settlements are vulnerable women in need of SRH services. They value the service and friendly behaviour of the SRH workers and, in return for the service, respect them, and feel happy to see them. For example, a female service promoter said, *“I like [working with] the pregnant women because when we visit them*, *they feel happy*. *They welcome us with a smile and say*, *‘sisters have come*!*’”*

#### Clients’ compliance

Clients’ compliance improves the motivation of the CTC SRH workers. Many SRH issues are highly stigmatized and hence take a lot of toll on the workers. Therefore, when they find that clients are following their instructions and visited the formal health facility after the referral, they feel rewarded. This is illustrated by the following quotation from a female service promoter: *“I feel good when the patients [clients] listen to us and take up the referral*, *and her husband follows our instructions too*… *It’s inspiring when they listen to what we have told them when they come [taking up the referral] here [clinic] following our words*. *Even mothers-in-law bring them here*! *Just a few days ago a mother-in-law brought her daughter-in-law here for menstrual regulation*. *They came here after our counseling*. *This is a great inspiration for us*.*”*

Referral uptake by their clients is very important to the CTC SRH workers because it has both monetary and non-monetary implications. This is evident in the following statement:

*“When the patients come*, *the recognition is good*. *If not*, *then we must hear [from the line manager] that “you are not doing fieldwork properly; not going to the field regularly*. *Therefore*, *the clients are not coming” … If I get recognition*, *then I get motivated to work better*. *[I work hard] thinking if I work better then I will get better appreciation*. *Sometimes*, *I feel disappointed because I’m working well*, *but if people don’t come*, *I can’t force them to come*.*”* (Health educator, female, 27-year-old).

## Discussion

The role of close-to-community (CTC) sexual and reproductive health (SRH) workers is vital to promote universalism, equality, non-discrimination, and inclusion in SRH services. They are useful to ensure that the SRH needs and rights of people living in low-income urban settlements are met. Here we investigated the factors that motivate or demotivate CTC SRH workers to continue their job in low-income urban settlements in Bangladesh.

Our findings reveal a range of factors that affect the motivation of CTC SRH workers in poor urban settlements in Bangladesh. As Herzberg and colleagues suggested in their two-factor theory on work motivation [24], the motivation of CTC SRH workers in low-income urban settlements in Bangladesh is influenced by a range of extrinsic and intrinsic factors (Table 2). These factors are related to their work, community, clients, personal, career, work environment, monetary incentives, and supervision. Similar findings are also reported by studies conducted elsewhere [25–30]. Factors identified in our study are in line with the existing models of work motivation, such as Herzberg’s two-factor models of work motivation [24,31] and the six primary needs or motives of the employees in a work setting proposed by Pareek [32].

Globally, CHWs mainly work voluntarily without or with very little financial incentive [21]. Our research participants included volunteer CTC workers and a mix of fully paid workers and workers paid for performance or commodity selling. Poor financial incentives, job insecurity, and lack of retirement benefits are identified as sources of dissatisfaction among both salaried and volunteer CTC SRH workers. It should be noted that CHWs mostly come from low-income backgrounds [21]. Giving time without or with limited financial benefit is a missed income opportunity for them. When there is a need for income or higher income, they might consider quitting their job and looking for alternative income-generating activities. The same is reported by many studies in Asia and Africa [33]. The commonly held belief is that paying CHWs is not sustainable [21]. But, when CHWs are in financial insecurity, they are more likely to quit and provide poor-quality service [33]. Recruiting new CHWs will be expensive because of the training and other resources need in the management [34]. Premature dropout of CHWs has a negative impact on the finances of the programme. Hence, this might also make a CHW programme unsustainable [34]. Our respondents unanimously expressed their frustration about the lack of or poor financial incentives and job insecurity. They believe that the amount of incentive they were receiving does not match their workload. In a country, where the unemployment rate is high, often people might take the job of CHWs to move up the career ladder or move to new employment. Herzberg noted that workers are more likely to be dissatisfied if they perceive unfairness in their financial incentives [24,31]. This dissatisfaction may work as a push factor [24,31] and may result in increased dropout of the CTC SRH workers. Therefore, we argue that CTC SRH workers should be adequately compensated for the time they devoted to the job.

Supportive, and regular, supervision was identified as another important extrinsic factor. More supportive approaches in supervision were mentioned as a positive factor in the literature as well [35]. Supervisors should act as mentors of the community SRH workers. They should monitor their performance and give appropriate feedback to increase the efficiency of their work [36]. Although supervision is considered an extrinsic factor, CTC SRH workers might consider it important to fulfill their career aspirations and growth needs. Hence, supportive, and regular supervision will positively affect the intrinsic factors/motivators of the CTC SRH workers.

A positive and trusting relationship between the health service providers, community, client, and the health system is important for the success of any health programme [37,38]. This kind of relationship maintains satisfaction and prevents dissatisfaction among the CTC SRH workers. We found that CTC SRH workers understand the importance of these relationships and strive to maintain and improve their relationships with the other stakeholders of the community SRH services.

We argue that the physical safety and security of the CTC SRH workers are given adequate attention by the community health organizations. Law and order in low-income urban settlements in LMICs are not often promising, as also reported by our respondents. A recent systematic review on the influence of the context on the performance of the CHWs reported that CHWs, particularly women, are concerned with their safety and this safety concern affects their motivation, and performance and often leads to dropout [33]. Therefore, community health organizations should develop strategies to minimize the CTC SRH workers’ physical risks in the community.

Along with the extrinsic or hygiene factors, a range of intrinsic factors or motivators were also found to drive the motivation of our respondents (Table 2). We found that CTC SRH workers find the motivation to continue their work when they believe or observe that their work has a positive impact on people’s lives and that the services they are providing are of good quality. Observing the positive impact of work is also reported as a strong motivator for the CHWs in the literature [21,39]. CHWs have varying levels of education [40] ranging from no education to a university degree in non-health fields in this current study. Therefore, in addition to appropriate training and orientation at recruitment, regular refresher training is vital to equip them with the necessary knowledge and skills to carry out the job. Training opportunities might also work as an incentive for the CTC SRH workers since this will help them to fulfill their career and personal growth aspirations.

There is also a spiritual dimension of motivation- satisfaction in serving people and serving God by serving the community. CTC SRH workers are often from the same or a similar community [33] and spend a great deal of time in the community. Hence, they understand the vulnerability of their clients- mostly women with very low socio-economic status. They take a great deal of pride and satisfaction, consequently motivation to continue, knowing that they are serving a vulnerable group in great need of SRH services. This spiritual dimension of the motivating factor was also evident in studies conducted elsewhere. For example, a study in Tanzania found that a desire to serve the God motivated the CHWs to become CHWs [41].

One of the most cited factors of motivation by our respondents is career growth and development opportunities. These factors also include the opportunity to improve knowledge and skills, and professional networking. This has also been reported consistently in the literature [21]. Career stagnation is demotivating. CHWs want to move up the career ladder. A hope to get a promotion might work as fuel for hard work and good service, but a lack of such hope might do just the opposite. However, to get a promotion, along with sincere work and quality performance, they also need to develop their skills and knowledge. Hence, to keep CTC SRH workers motivated, in addition to promotion opportunities there should be training opportunities for the CTC SRH workers to improve their knowledge and skill in community SRH services. CHWs view gaining new knowledge and skills as a path to better employment or promotion opportunities in the same organization [21].

Our respondents unanimously identified recognition of their services from the client, community, and higher-level authority as an important determinant of their motivation. Evidence suggests that lack of support and respect from the higher-level authority and or the community negatively affect the motivation and performance of the CHWs [38].

One of the main jobs of the CTC SRH workers in low-income urban settlements in Bangladesh is to refer women with SRH service needs, including menstrual regulation, to the clinic they are affiliated with. Referral uptake by the clients is considered a key performance indicator of the CTC SRH workers. We found that successful referrals to health facilities work as a strong motivator among CTC SRH workers in Bangladesh. CHW literature also indicates successful referral to a health facility as a complementary incentive to the CHWs [42]. However, CTC SRH workers in Bangladesh operate in a pluralistic setting, where there is stiff competition between different formal, informal, and private providers. The majority of our respondents reported difficulty in achieving the referral targets. Further research is needed to understand the factors affecting referral uptake by women with SRH needs in low-income urban settlements in Bangladesh.

It is important to keep CTC SRH workers motivated to prevent premature dropout. A high dropout is related to poor programme performance and higher programme costs. An important theory to understand what motivates an individual in a work condition is the Maslow’s Hierarchy of Needs [43]. Extrinsic factors identified in our study lie in the bottom tiers of the Maslow’s Hierarchy of Needs, whereas the motivators or intrinsic factors can be placed in the upper tiers. It is suggested that unless an individual’s basic needs have been met, higher levels in the hierarchy are of no relevance [43,44]. A study on nurses in New Zealand also found that most cited concerns are related to hygiene factors [45]. Therefore, organizations need to make sure that these hygiene factors are adequately met. Unmet hygiene factors might demotivate the CTC SRH workers and cause them to work less hard.

Our studies included both salaried and volunteer CTC SRH workers and they were recruited from both the government and NGO sectors. However, we did not compare motivation between these groups. It would be interesting to know how these CTC SRH worker groups differ in terms of their work motivation. Further study should focus on that.

## Conclusion

A range of extrinsic/hygiene factors and intrinsic factors/motivators affect the motivation of CTC SRH workers in low-income urban settlements in Bangladesh. Commonly cited reasons for dissatisfaction among the CTC SRH workers are poor salaries or other financial incentives, lack of job security, and absence of supportive supervision. While, the motivators include witnessing the positive impact of work, promotion, developing new knowledge and skills, and successful referral among others. Community health programmes are vital in promoting universalism and equity in the health of the population. CHWs are at the heart of any community health programmes. To prevent premature dropout and ensure quality community SRH services employers of the CTC SRH workers should give adequate emphasis on meeting both hygiene factors and motivators.

## Abbreviations

CTC: Close-to-Community
SRH: Sexual and Reproductive Health
CHW: Community Health Worker
LMICs: Low and middle-income countries
NGO: Non-Governmental Organization

## References

[1] Streatfield PK, Karar ZA. Population challenges for Bangladesh in the coming decades. J Health Popul Nutr. 2008;26(3):261–72. . 18831223 PMC2740702

[2] Adams AM, Islam R, Ahmed T. Who serves the urban poor? A geospatial and descriptive analysis of health services in slum settlements in Dhaka, Bangladesh. Health policy and planning. 2015;30 Suppl 1:i32–45. Epub 2015/03/12. doi: 10.1093/heapol/czu094 ; PubMed Central PMCID: PMC4353891. 25759453 PMC4353891

[3] Theobald S, Hawkins K, Kok M, Rashid S, Datiko DG, Taegtmeyer M. Close-to-community providers of health care: increasing evidence of how to bridge community and health systems. Human resources for health. 2016;14(1):32. doi: 10.1186/s12960-016-0132-9 27250338 PMC4890328

[4] Gilmore B, McAuliffe E. Effectiveness of community health workers delivering preventive interventions for maternal and child health in low- and middle-income countries: a systematic review. BMC public health. 2013;13:847. Epub 2013/09/17. doi: 10.1186/1471-2458-13-847 ; PubMed Central PMCID: PMC3848754. 24034792 PMC3848754

[5] McPake B, Edoka I, Witter S, Kielmann K, Taegtmeyer M, Dieleman M, et al. Cost-effectiveness of community-based practitioner programmes in Ethiopia, Indonesia and Kenya. Bull World Health Organ. 2015;93(9):631–9A. Epub 2015/08/03. doi: 10.2471/BLT.14.144899 . 26478627 PMC4581637

[6] Vallieres F, Hyland P, McAuliffe E, Mahmud I, Tulloch O, Walker P, et al. A new tool to measure approaches to supervision from the perspective of community health workers: a prospective, longitudinal, validation study in seven countries. BMC health services research. 2018;18(1):806. Epub 2018/10/24. doi: 10.1186/s12913-018-3595-7 ; PubMed Central PMCID: PMC6196473. 30348147 PMC6196473

[7] Glenton C, Colvin CJ, Carlsen B, Swartz A, Lewin S, Noyes J, et al. Barriers and facilitators to the implementation of lay health worker programmes to improve access to maternal and child health: qualitative evidence synthesis. Cochrane Database Syst Rev. 2013;10:CD010414. Epub 2013/10/09. doi: 10.1002/14651858.CD010414.pub2 . 24101553 PMC6396344

[8] Ahmed SM, Hossain MA, RajaChowdhury AM, Bhuiya AU. The health workforce crisis in Bangladesh: shortage, inappropriate skill-mix and inequitable distribution. Human resources for health. 2011;9(1):3. doi: 10.1186/1478-4491-9-3 21255446 PMC3037300

[9] Nkonki L, Cliff J, Sanders D. Lay health worker attrition: important but often ignored. Bulletin of the World Health Organisation. 2011;89(12):853–928. Epub 24 October 2011. doi: 10.2471/BLT.11.087825 22271950 PMC3260896

[10] Kironde S, Klaasen S. What motivates lay volunteers in high burden but resource-limited tuberculosis control programmes? Perceptions from the Northern Cape province, South Africa. Int J Tuberc Lung Dis. 2002;6(2):104–10. Epub 2002/04/05. . 11931408

[11] MacQueen KM, McLellan E, Metzger DS, Kegeles S, Strauss RP, Scotti R, et al. What is community? An evidence-based definition for participatory public health. Am J Public Health. 2001;91(12):1929–38. Epub 2001/12/01. doi: 10.2105/ajph.91.12.1929 ; PubMed Central PMCID: PMC1446907. 11726368 PMC1446907

[12] Abbey M, Bartholomew LK, Nonvignon J, Chinbuah MA, Pappoe M, Gyapong M, et al. Factors related to retention of community health workers in a trial on community-based management of fever in children under 5 years in the Dangme West District of Ghana. Int Health. 2014;6(2):99–105. Epub 2014/02/18. doi: 10.1093/inthealth/ihu007 . 24532651

[13] Bonenberger M, Aikins M, Akweongo P, Wyss K. The effects of health worker motivation and job satisfaction on turnover intention in Ghana: a cross-sectional study. Human resources for health. 2014;12:43. Epub 2014/08/12. doi: 10.1186/1478-4491-12-43 ; PubMed Central PMCID: PMC4130118. 25106497 PMC4130118

[14] Dugani S, Afari H, Hirschhorn LR, Ratcliffe H, Veillard J, Martin G, et al. Prevalence and factors associated with burnout among frontline primary health care providers in low- and middle-income countries: A systematic review. Gates open research. 2018;2:4. Epub 2018/07/10. doi: 10.12688/gatesopenres.12779.3 ; PubMed Central PMCID: PMC6030396. 29984356 PMC6030396

[15] Chimwaza W, Chipeta E, Ngwira A, Kamwendo F, Taulo F, Bradley S, et al. What makes staff consider leaving the health service in Malawi? Human resources for health. 2014;12:17. Epub 2014/03/20. doi: 10.1186/1478-4491-12-17 ; PubMed Central PMCID: PMC3974437. 24641840 PMC3974437

[16] Alhassan RK, Spieker N, van Ostenberg P, Ogink A, Nketiah-Amponsah E, de Wit TFR. Association between health worker motivation and healthcare quality efforts in Ghana. Human resources for health. 2013;11(1):37. doi: 10.1186/1478-4491-11-37 23945073 PMC3765920

[17] Dieleman M, Cuong PV, Anh LV, Martineau T. Identifying factors for job motivation of rural health workers in North Viet Nam. Human resources for health. 2003;1(1):10. Epub 2003/11/14. doi: 10.1186/1478-4491-1-10 ; PubMed Central PMCID: PMC280735. 14613527 PMC280735

[18] Kok MC, Dieleman M, Taegtmeyer M, Broerse JE, Kane SS, Ormel H, et al. Which intervention design factors influence performance of community health workers in low-and middle-income countries? A systematic review. Health policy and planning. 2015;30(9):1207–27. doi: 10.1093/heapol/czu126 25500559 PMC4597042

[19] Kok MC, Kane SS, Tulloch O, Ormel H, Theobald S, Dieleman M, et al. How does context influence performance of community health workers in low-and middle-income countries? Evidence from the literature. Health Research Policy and Systems. 2015;13(1):13. doi: 10.1186/s12961-015-0001-3 25890229 PMC4358881

[20] Pallas SW, Minhas D, Perez-Escamilla R, Taylor L, Curry L, Bradley EH. Community health workers in low- and middle-income countries: what do we know about scaling up and sustainability? Am J Public Health. 2013;103(7):e74–82. Epub 2013/05/18. doi: 10.2105/AJPH.2012.301102 . 23678926 PMC3682607

[21] Bhattacharyya K, Winch P, LeBan K, Tien M. Community Health Worker Incentives and Disincentives: How they affect motivation, retention, and sustainability. Arlington, Virginia: Basic Support for Institutionalizing Child Survival Project (BASICS II) for the United States Agency for International Development, 2001 October. Report No.

[22] Mahmud I, Chowdhury S, Siddiqi BA, Theobald S, Ormel H, Biswas S, et al. Exploring the context in which different close-to-community sexual and reproductive health service providers operate in Bangladesh: a qualitative study. Human resources for health. 2015;13(1):51. doi: 10.1186/s12960-015-0045-z 26323508 PMC4556024

[23] Ritchie J, Spencer L. Chapter 9: Qualitative data analysis for applied policy research. In: Burgess RG, Bryman A, editors. Analyzing qualitative data. London; New York: Routledge; 1994. p. 173–94.

[24] Herzberg F, Mausner B, Snyderman B. The motivation to work, 2nd ed. Oxford, England: John Wiley; 1959. xv, 157–xv, p.

[25] Bhattacharyya K, Winch P, LeBan K, Tien M. Community health worker incentives and disincentives: how they affect motivation retention and sustainability. 2001.

[26] Tripathy JP, Goel S, Kumar AM. Measuring and understanding motivation among community health workers in rural health facilities in India-a mixed method study. BMC health services research. 2016;16(1):366. doi: 10.1186/s12913-016-1614-0 27507034 PMC4977615

[27] Jigssa HA, Desta BF, Tilahun HA, McCutcheon J, Berman P. Factors contributing to motivation of volunteer community health workers in Ethiopia: the case of four woredas (districts) in Oromia and Tigray regions. Human resources for health. 2018;16(1):57. doi: 10.1186/s12960-018-0319-3 30409189 PMC6225677

[28] Greenspan JA, McMahon SA, Chebet JJ, Mpunga M, Urassa DP, Winch PJ. Sources of community health worker motivation: a qualitative study in Morogoro Region, Tanzania. Human resources for health. 2013;11(1):52. doi: 10.1186/1478-4491-11-52 24112292 PMC3852396

[29] Thu NTH, Wilson A, McDonald F. Motivation or demotivation of health workers providing maternal health services in rural areas in Vietnam: findings from a mixed-methods study. Human resources for health. 2015;13(1):91. doi: 10.1186/s12960-015-0092-5 26626015 PMC4667451

[30] Berman PA, Gwatkin DR, Burger SE. Community-based health workers: head start or false start towards health for all? Social Science & Medicine. 1987;25(5):443–59. doi: 10.1016/0277-9536(87)90168-7 3118477

[31] Herzberg F. One More Time: How Do You Motivate Employees? Harvard Business Review. 2003;(January 2003). 12545925

[32] Pareek U. Motivational Analysis of Organizations- Behavior (MAO-B). 2007. In: The Pfeiffer Book of Successful Leadership Development Tools [Internet]. San Fransisco: Pfeiffer. Available from: https://learning.oreilly.com/library/view/the-pfeiffer-book/9780470181812/ch30.html.

[33] Kok MC, Kane SS, Tulloch O, Ormel H, Theobald S, Dieleman M, et al. How does context influence performance of community health workers in low- and middle-income countries? Evidence from the literature. Health Research Policy and Systems. 2015;13(1):13. doi: 10.1186/s12961-015-0001-3 25890229 PMC4358881

[34] Alam K, Khan JA, Walker DG. Impact of dropout of female volunteer community health workers: an exploration in Dhaka urban slums. BMC health services research. 2012;12:260. Epub 2012/08/18. doi: 10.1186/1472-6963-12-260 ; PubMed Central PMCID: PMC3464156. 22897922 PMC3464156

[35] Ndima SD, Sidat M, Ormel H, Kok MC, Taegtmeyer M. Supervision of community health workers in Mozambique: a qualitative study of factors influencing motivation and programme implementation. Human resources for health. 2015;13(1):63. doi: 10.1186/s12960-015-0063-x 26323970 PMC4556309

[36] Mathauer I, Imhoff I. Health worker motivation in Africa: the role of non-financial incentives and human resource management tools. Human resources for health. 2006;4(1):24. doi: 10.1186/1478-4491-4-24 16939644 PMC1592506

[37] Gilson L. Trust and the development of health care as a social institution. Social science & medicine (1982). 2003;56(7):1453–68. Epub 2003/03/05. doi: 10.1016/s0277-9536(02)00142-9 . 12614697

[38] Kok MC, Broerse JEW, Theobald S, Ormel H, Dieleman M, Taegtmeyer M. Performance of community health workers: situating their intermediary position within complex adaptive health systems. Human resources for health. 2017;15(1):59. Epub 2017/09/04. doi: 10.1186/s12960-017-0234-z ; PubMed Central PMCID: PMC5581474. 28865471 PMC5581474

[39] Wollinka O, Keeley E, Burkhalter BR, Bashir N. Hearth Nutrition Model: Applications in Haiti, Vietnam, and Bangladesh. Arlington, Virginia: Basic Support for Institutionalizing Child Survival (BASICS) Project, 1997.

[40] Lehmann U, Sanders D. Community health workers: What do we know about them? The state of the evidence on programmes, activities, costs and impact on health outcomes of using community health workers. Geneva: Evidence and Information for Policy, Department of Human Resources for Health, World Health Organization, 2007.

[41] Mpembeni RN, Bhatnagar A, LeFevre A, Chitama D, Urassa DP, Kilewo C, et al. Motivation and satisfaction among community health workers in Morogoro Region, Tanzania: nuanced needs and varied ambitions. Human resources for health. 2015;13:44. Epub 2015/06/06. doi: 10.1186/s12960-015-0035-1 ; PubMed Central PMCID: PMC4458000. 26044146 PMC4458000

[42] Colvin CJ. What Motivates Community Health Workers? Designing Programs that Incentivize Community Health Worker Performance and Retention In: Perry H, Crigler L, editors. Developing and Strengthening Community Health Worker Programs at Scale: A Reference Guide and Case Studies for Program Managers and Policymakers: The Maternal and Child Health Integrated Program (MCHIP), United States Agency for International Development (USAID); 2014. p. 11.1-.6.

[43] Maslow AH. Motivation and personality: Prabhat Prakashan; 1981.

[44] Benson SG, Dundis SP. Understanding and motivating health care employees: integrating Maslow’s hierarchy of needs, training and technology. Journal of nursing management. 2003;11(5):315–20. Epub 2003/08/22. doi: 10.1046/j.1365-2834.2003.00409.x . 12930537

[45] Jamieson I, Kirk R, Wright S, Andrew C. Generation Y New Zealand Registered Nurses’ views about nursing work: a survey of motivation and maintenance factors. Nursing open. 2015;2(2):49–61. Epub 2015/04/07. doi: 10.1002/nop2.16 ; PubMed Central PMCID: PMC5047315. 27708801 PMC5047315

